# Double layer capacitors in dye sensitized solar cells with large charge and energy storage capacity and controlled shape of output voltage signals

**DOI:** 10.1371/journal.pone.0298776

**Published:** 2024-05-28

**Authors:** Susana Vargas, Domingo Rangel, Juan Carlos Gallegos, Rogelio Rodríguez

**Affiliations:** Centro de Física Aplicada y Tecnología Avanzada, Universidad Nacional Autónoma de México, Querétaro, México; K Ramakrishnan College of Technology, INDIA

## Abstract

The output signals in natural dyes-based solar cells (DSSC) can be either rising or decaying depending on the type of ions present in the system; these ions called added ions, are introduced by the additives: mordant and brighteners. The photon-dye interaction produces electrons, which eventually reach the electrode giving place to a superficially charged electrode in contact with an electrolyte where are the added ions. This combination produces, automatically, an electrical double-layer EDL structure which has important effects on the performance of the system: a) the added ions control, to a large extent, the initial shape of the output signal, giving rise to rising or decaying profiles; b) it is possible to store large amounts of energy and charge at high electric fields. This structure is found in many other systems that have a surface charged in contact with an electrolyte like piezoelectric materials in human body. This assertion was supported by determining important parameters such as the force between charged surfaces on both sides of the interface, the charge density, the energy density, and the capacitance. The Debye length has very small values then, many important quantities depend on this; it is possible to obtain large values for energy U_DL_ ~ 3.6x10^5^ Jm^-3^ and charge density ρ_DL_ ≈ 1.1x10^7^ Cm^-3^ for double layer capacitors; these values are orders of magnitude larger than the corresponding values for electrostatic capacitors: U_elec_ ≈ 4.5x10^-3^ Jm^-3^ and ρ_elec_ ≈ 1.2 Cm^-3^. A non-linear model was also developed to fit unstable oscillations found in the output profiles produced by abrupt lighting.

## Introduction and theoretical considerations

From Gratzel in 1991 to these days [1], significant advances have been reached in the different parts that make up the structure of the cell [2, 3]. Among the parts that make up the cell, the electrolyte is essential since it is in physical contact with both electrodes, and it is where ionic transport takes place [4]. The charge transport in the electrolyte is by ions; some of them move toward the electrodes to form the double-layer [5, 6], while others, such as the redox couple [7], are responsible for transporting electrons from the electrode toward the excited dye [8]. Due to the importance of ionic transport in cell performance, a brief description of the processes that occur in DSSC is included:

- the anode was covered with a mesoporous impregnated (sensitized) with the dye (TiO_2_-S) on both internal and external surfaces.
- the light impinges the sensitized dye (in ns) (TiO_2_-S + hν → TiO_2_-S*), emitting electrons to the conduction band of the mesoporous (in ps) (TiO_2_-S* → TiO_2_-S^+^ + e^-^), which circulates through the external circuit, reaching the cathode.
- the exited dye molecules are stabilized through chemical bonds with the mesoporous; ions of polyvalent metals carry out this union.
- the electron is captured at the cathode by I_3_^-^ which is reduced to I^-^ and transported to the dye (in ms-s) ((1/2)I_3_^-^ + e^-^ → (3/2)I^-^).
- the arriving electron reacts with the excited dye reducing it (in μs-ms) (TiO_2_-S^+^ + e^-^ → TiO_2_-S).
- the dye is regenerated by reacting with the electron captured by I_3_^-^ and transported by I^-^ to its place (in μs) (TiO_2_-S^+^ + (3/2)I^-^ → TiO_2_-S + (1/2)I_3_^-^)
- I_3_^-^ returns to the cathode to continue the electron capture (ms-s) (I_3_^-^ + 2e^-^ → 3I^-^); the role of I_3_^-^ is to capture the electron at the cathode, and the role of I^-^ is to transport the electron to the dye to reduce it. This reaction is essential since it can modify the efficiency of the cell because it is limited to the surface of the mesoporous.
- the carbon layer on the cathode acts as a catalyst to improve electron capture by I_3_^-^.

This work has two basic purposes understanding and predicting the initial shape of the output signals, and estimating the typical values that can be obtained for the double layer capacitors; these values were obtained based on those reported in the literature for basic quantities like the Debye length. The high values obtained for these quantities, such as stored energy and charge, are essentially due to the small value of the Debye length.

### Double-Layer formation, ionic transport, charge accumulation

When light impinges the dye, the emitted electrons eventually reach the cathode after a time τ_e_, charging it and producing an electric field $E_{e}=\frac{n_{e}q_{e}}{\varepsilon A}$ and a voltage V_e_; this field is proportional to the charge accumulated in a very thin layer on the electrode surface of the order of λ_e-e_ < 0.1 Å; here, n_e_q_e_ = en_e_, n_e_, q_e_, ε and A are the number and charge of the electrons, the dielectric permittivity of acetonitrile and the surface area; the thickness of the electron layer on the electrode λ_e-e_ is very small because of the tiny size of electrons and the fact that they can be transported out of the electrode through the interface by the redox couple (faradaic transport) leaving only a single layer on the metal surface and preventing the formation of a multi-layer structure. The field E_e_ moves the ions in the electrolyte toward the electrode of opposite charge and accumulates near it forming, in a time τ_i_, a diffuse charge layer of thickness λ; this accumulation of ions in EDL produces the field $E_{i}=\frac{n_{i}q_{i}}{\varepsilon A}$ and a voltage V_i_; the field E_i_ is proportional to the accumulated charge n_i_q_i_ (= n_i_z_i_e), where n_i_, q_i_, and z_i_ are the number, charge, and valence of the ions; because the fields and voltages are in opposition, the output voltage V_o_ is giving by V_o_ = V_e_—V_i_; according to this equation, V_e_ increases the output voltage, while V_i_ reduces it.

When the ions start moving towards EDL, the first layer that begins to fill is the Stern layer which is the layer closest to the interface [9]. The charge accumulation in the Stern layer involves high internal energies due to intense repulsive forces between charges of the same sign; there is a strong restriction imposed to the charge separation distance: there is a minimum approach distance d_min_ between charges, which limits the maximum concentration of charge that can be accumulated in a finite space; then, the filling of the Stern layer ends when the distance between ions reaches the value d_min_; the rest of ions go to the next second layer, which is filled also respecting d_min_; this process continues filling successive layers as close as possible to the interface; it is in this way that the EDL is formed: the Stern layer and a few adjacent layers form the EDL which has a thickness λ and is in contact with the interface; the restriction imposed by d_min_ produces non-uniform ions distribution with higher density near the charged surface, and with a diffuse structure (Bockris-Devanathan-Mueller model). The restriction imposed by d_min_ and the strong and attractive force between charged surfaces at both sides of the interface have interesting effects: a) produce a tight and orderly packing of ions in the diffuse layer, and b) the strong interfacial attractive force between charged surfaces stabilizes the whole EDL structure. Even when both phases are in physical contact with each other, the interface itself prevents the transport of ions through it (a non-faradaic process), avoiding positive and negative charges from mixing and canceling each other, but rather remaining separate forming a system of two charged surfaces separated by a dielectric; this structure resembles a capacitor, a particular type of capacitor called double-layer capacitor DLC or Helmholtz double-layer capacitor HDLC. This process produces a double-layer formation on both sides of the interface: on one side, an electrode containing a thin layer of electrons with a thickness λ_e-e_, while on the other side, an electrolyte containing a diffuse layer of ions with thickness λ and in contact with the interface; the diffuse layer is made up of several layers, the first one and closest to the interface is the Stern layer which consists of ions strongly adsorbed on the interface due to strong, attractive electrostatic forces with the electrons in the electrode; in this layer, there are also solvent molecules which solvate the ions. The second layer, adjacent to the Stern layer, is composed of solvated ions and counterions and attracted electrostatically to the charge of the electrode with the corresponding screening effect produced by the Stern layer; in contract, the ions in the first layer are firmly anchored to the interface, in the second one these are loosely associated with the electrode and they can move in the fluid under the influence of electric interactions and thermal effects.

### Shapes of the output voltage profiles

**Fig 1:**
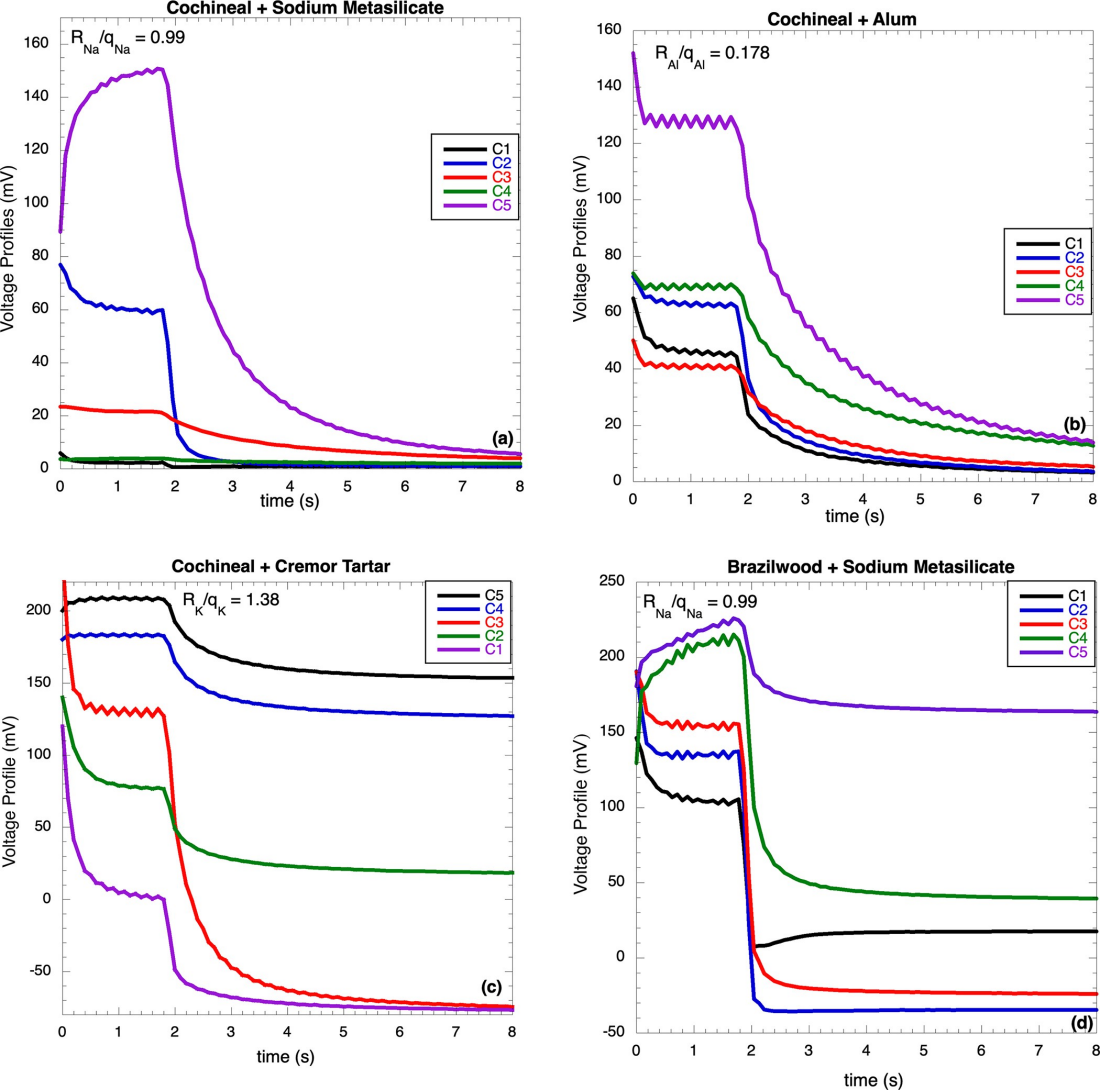
Voltage profiles of a) cochineal-metasilicate, b) cochineal-alum, c) cochineal-CT, d) brazilwood-metasilicate; the profiles were shifted for clarity.

One of the most notable characteristics of the output voltage profiles is their initial shape; this is important because the cells are connected to different devices and the characteristics of the output signals can affect these; sometimes the shape is a rising exponential, while others times it is a decaying. This is produced by a competition between the arrival times of electrons τ_e_ and ions τ_i_ to their corresponding destinations. The arrival time τ_e_ is practically constant because the charges are always electrons at the same concentration at constant illumination, while the time τ_i_ depends on the size, charge, and concentration of various types of added ions introduced into the cell by the additives (mordants and brighteners). The added ions can be divided into two categories according to their physical characteristics: slow and fast ions. The slow ions are those that have a large size, low charge, and high concentration; these move at a small velocity (τ_i_ > τ_e_), producing a considerable delay in the formation of EDL without any affectation to the deposition rate of electrons to the electrode, which increases the output voltage resulting in a rising profile. On the other hand, fast ions have a small size, high charge, and low concentration; they move rapidly (τ_i_ < τ_e_) forming in short times EDL, which reduces the output voltage, resulting in a decaying profile. This variety of rising and decaying profiles can be seen in Fig 1A–1D; for example, in Fig 1D the dye is brazilwood and the brightener sodium metasilicate; from this set, two profiles are rising and three decaying; consequently, this effect can only be produced by concentration. From this discussion, it is possible to see that τ_i_ depends on the ratio R_i_/q_i_, where R_i_ is related to the viscous force and q_i_ to the electric force; it is necessary to include the dependence of τ_i_ with the concertation n_i_. This is a peculiar characteristic of the ionic transport; due to the large size of the ions, their transport is modeled as a series of jumps to neighboring sites of the same size; however, when there are a large number of ions close to the ion that is jumping, they block the jump reducing the ionic transport; this is called “obstruction effect”; this means that the ionic conduction, i.e., τ_i_, depends on the concentration n_i_; this effect can be taken into account by introducing a factor n_i_ in the numerator in the expression for τ_i_; then, τ_i_ ~ n_i_R_i_/q_i_; because the ions were introduced at concentrations C1-C5:

$$\tau_{i}∼C_{i}R_{i}/q_{i}$$ (1)

**Fig 2:**
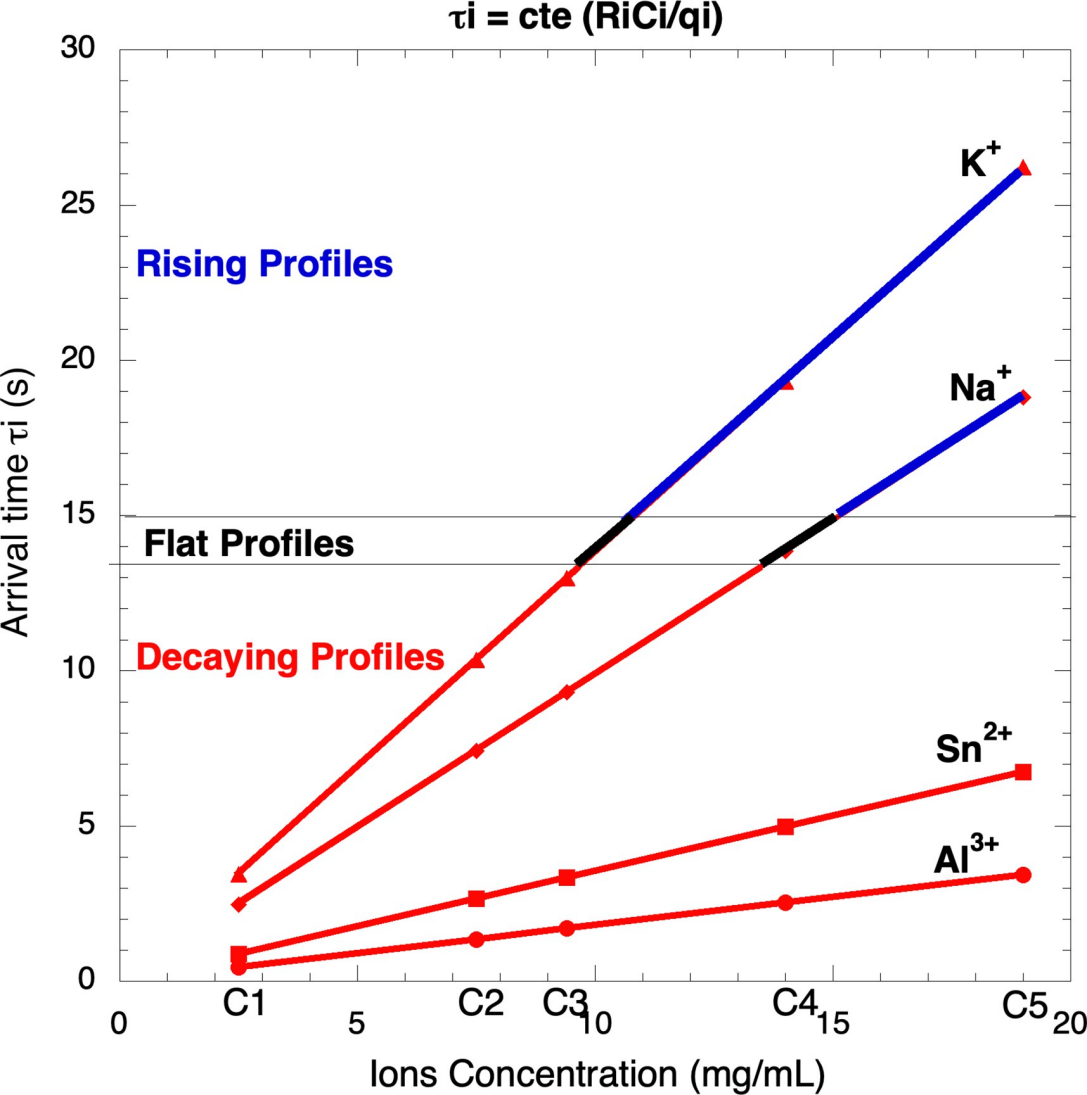
Plot of the equation τ_i_ ~ (R_i_/q_i_)C_i_ for four cations (Al^3+^, Sn^2+^, Na^+^, K^+^) at different concentrations C1-C5. Fast ions (Al^3+^, Sn^2+^) produce decaying profiles (red); slow ions (Na^+^, K^+^) can produce either rising (blue) or decaying (red) profiles depending on concentration.

With this equation, it is possible to determine the arrival times of different types of ions to their destinations, and to predict the shape of the output profiles. In a time-concentration space, this equation corresponds to a straight line passing through the origin with a slope of R_i_/q_i_: for each type of ion, there is a straight line, as shown in Fig 2.

A typical capacitor is a device that stores energy as electric field in a finite region of space; this is generally composed of two metal plates that delimit the electric field and are separated by a dielectric material. This device is governed by the relationship Q = CV, where Q, V, and C are charge, potential, and capacitance; the charge in a capacitor consists of an excess of electrons in one plate and a deficiency of them in the other and a polarized dielectric in between. On the other hand, the DLC has no metallic borders that delimit the electric field, but in its place is the double-layer structure which, by itself, plays that role: the double-layer does not produce a well-defined edge but a diffuse one; DLC is not a typical electrostatic capacitor. The double layer capacitor has received different names; double-layer capacitor DLC, Helmholtz double-layer capacitor HDLC or supercapacitor. The charge is accumulated at different speeds on the electrode and EDL: fast at the beginning and slower at the end; this is because the traveling charges have to overcome the field produced by the charges already accumulated. The rate at which the charges are being deposited depends on the charges already deposited: dq/dt ∞ -q; the solution of this differential equation assuming the q = 0 at t = 0 is q = q_o_(1-e^-t/τ^); because the charge and the voltage are proportional for electrons and ions:

$$V_{e}=V_{oe}[1‐e^{‐t/\tau_{e}}]+V_{eDC}\;V_{i}=V_{oi}[1‐e^{‐t/\tau_{i}}]+V_{iDC}$$ (2A, 2B)

These equations describe the filling kinetics of electrons at the electrode and ions at EDL. V_e_ and V_i_ are part of the output voltage V_o_ = V_e_−V_i_. There are two possible situations in the filling kinetics: for slow ions (τ_i_ > τ_e_), they move so slowly that there is a delay in the EDL formation leaving unaffected the rate at which electrons are deposited on the electrode, which increases the output voltage; then, V_o_ can be approximated by V ≈ V_e_, and from Eq 2A, $V=V_{\mathrm{oe}}[1‐e^{‐t/\tau_{e}}]$ resulting in a rising profile:

$$V=V_{o}[1‐e^{‐t/\tau }]+V_{\mathrm{DC}}\;\mathrm{slow}\;\mathrm{ions}\;(\tau_{i}>\tau_{e})$$ (3A)

For fast ions (τ_i_ < τ_e_), they form the EDL rapidly; however, a threshold voltage V_oe_ is required to initiate the ionic transport and once initiated, they quickly reach EDL while V_oe_ remains practically constant; then, using Eq 2B $V=V_{\mathrm{oe}}‐V_{i}={(V}_{oe}-V_{oi}{)+V}_{\mathrm{oi}}e^{-\frac{t}{\tau_{i}}}$; in this case, the output profile is decaying:

$$V=V_{o}e^{‐t/\tau }+V_{\mathrm{DC}}\;\mathrm{fast}\;\mathrm{ions}\;(\tau_{i}<\tau_{e})$$ (3B)

Eqs 3A and 3B correctly describe the shape of the output profiles; however, two exponentials are required to fit the experimental data due to the presence of different types of added ions:

$$V(t)=V_{o1}e^{‐t/\tau_{1}}+V_{o2}e^{‐t/\tau_{2}}+\mathrm{BL}$$ (4A)

$$V(t)=V_{o1}(1‐e^{‐t/\tau_{1}})+V_{o2}(1‐e^{‐t/\tau_{2}})+\mathrm{BL}$$ (4B)

where τ_1_, τ_2_ are the relaxation times, V_o1_, V_o2_ the voltage amplitudes, and BL the baseline. It is necessary to include in these equations a term to fit the oscillations. The times τ_1_ and τ_2_ are related, but not equal, to the arrival times τ_e_ and τ_i_.

### Numerical estimation of DSSC parameters

It has been mentioned that the double-layer structure can store large amounts of energy and charge at a high electric field; to support this assertion, the values of important characteristic parameters associated with the performance of DSSC will be numerically estimated: stored energy, stored charge, electric field, and capacitance; the numerical estimation of these parameters was based on reported values of some basic quantities characteristic of the double-layer capacitor structure like: double-layer thickness λ, Stern layer thickness λ_SL_, the distance between charged particles d_min_ in a charge accumulation. The separation distance δ between charged surfaces is δ = λ_SL_ + λ_e-e_ ≈ λ_SL_; this distance is essentially given by the Stern distance λ_SL_ which is of the order of λ_SL_ ≈ 0.2 nm ≈ 2.0x10^-10^ m [12]; this very small distance produces an intense attractive force F_I_; this intense, interfacial force stabilizes the DLC structure causing accommodating ions in a tight, orderly manner very near to saturation conditions.

The basic quantities λ, λ_SL_ and d_min_ are reported in the literature for a wide variety of systems and different experimental conditions and have been grouped, in all cases, as ranges of numerical values associated; some of these values have been measured experimentally, while others have been obtained based on some theoretical model like the Debye-Huckel model [10]. From each of these ranges, a value was selected approximately in the middle range and assigned to the corresponding basic quantity; in this way, this value is representative of the basic quantity in question [10]. The estimated values of DSSC parameters obtained by this methodology provide a clearer idea of the potential applications of DLC. It is possible to mention, in advance, that the DLCs have a very appropriate structure to significantly increase the charge and energy that can be stored in a DL without losing its stability; an additional advantage of this type of structure is its extraordinarily small size which is of the order of Aλ where λ is the Debye length. These characteristics open up many interesting potential applications in different fields of science and technology: in the design of new, very high-performance batteries, in the analysis of body piezoelectric signals, in the development of a new generation of medical equipment, etc.

As mentioned, a charged surface in contact with an electrolyte forms a DLC where the charge, energy, electric field, and capacitance are confined to a small volume Aλ, being λ the double-layer thickness:

$$\lambda =\sqrt{\frac{\varepsilon \mathrm{kT}}{n_{o}{z^{2}e}^{2}}}\;\mathrm{and}\;\kappa ={\left(\lambda \right)}^{-1}=\sqrt{\frac{n_{o}{z^{2}e}^{2}}{\varepsilon \mathrm{kT}}}$$ (5)

The values of the basic quantities have a direct effect on the performance of the cell: the distance between charged surfaces δ ≈ λ_SL_ provides information with respect to the stability of the whole DLC structure; the thickness λ of DL is important because electric field, charge, energy, are limited to this distance, the area A of the charged surfaces contribute to the force between them. Once numerical values have been assigned to the basic quantities, the values of the parameters can be calculated. These parameters allow to estimate how much energy and charge can be stored in DLC; typical values of standard capacitors are reported in Table 1 for comparison purposes.

**Table 1:** Typical values for double layer and electrostatic capacitors.

	Double Layer Capacitor	Electrostatic Capacitor
Energy (J)	1.4x10^-7^ J	1.8x10^-9^ J
Energy Density	3.6x10^5^ J/m^3^	4.5x10^-3^ J/m^3^
Electric Field	3.4x10^7^ V/m	3.7x10^6^ V/m
Electric Charge	4.5x10^-6^ C	4.9x10^-7^ C
Charge Density	1.1x10^7^ C/m^3^	1.2 C/m^3^
Capacitance	1.3x10^-4^ F	1.3x10^-10^ F
Interface Force	9.09x10^2^ N	---
Volume	4x10^-13^ m^3^	4x10^-7^ m^3^

Numerical values will be assigned to the basic quantities. It has been reported values of λ and κ (= λ^-1^) for different electrolytes at different concentrations and valences in aqueous solutions at 25°C [11]; from this published data, the values of λ are in the range λ ∈ [2.5x10^-10^, 1.0x10^-8^] m, and for its inverse κ = λ^-1^, κ ∈ [1.0x10^8^, 4.0x10^9^] m^-1^; then, values in the middle range were selected for λ and κ: λ = 10^−9^ m and κ = 10^9^ m^-1^. Regarding the cell geometry, typical values were chosen for the area and separation distance: a typical area of 2x2 cm^2^ was chosen, A = 2x2 cm^2^ = 4x10^-4^ m^2^ and a distance d = 1 mm = 10^−3^ m; the electric permittivity of acetonitrile ε_acetonitrile_ = 3.32 x 10^−10^ Fm^-1^. For the Stern layer, because this is built from solvated ions, the thickness of this layer has an average value [12] of 0.2 nm, i.e., λ_SL_ = 2.0x10^-10^ m. The Stern layer is the layer that contributes the most to the interfacial force between the charged surfaces.

The accumulation of electrons in the electrode, although they are subject to the criterion of the minimum distance between charges, d_min_, they generally form only a very thin single layer with a thickness of around λ_e-e_ ≤ 0.1 Å. The charges accumulated in the electrode and the electrolyte (Stern layer) are closely packed due mainly to the intense attraction force between electrons and ions; the ions in the Stern layer behave much like a two-dimensional ideal gas, accommodating an ion in an area of 10 nm^2^; this corresponds to a surface charge density σ_DL_ of $\sigma_{\mathrm{DL}}=\frac{1\;\mathrm{ion}}{10\;{\mathrm{nm}}^{2}}=1.6x10^{‐2}\frac{C}{m^{2}}$ [13]; the charges in this monolayer are separated by a distance d_min_ = √(10 nm^2^) = 3.16 nm. Then, there are two characteristic distances: ion-ion (d_min_ = 3.16 nm) and electron-ion (λ_SL_ = 0.5 nm); the ion-ion distance is approximately equal to electron-electron for monovalent ions.

Table 1 reports typical values for double layer and electrostatic capacitors; as can be seen, the energy and charge densities are several orders of magnitude higher with respect to the electrostatic one.

The electric potential Ψ and the electric field E are possibly the more measured and calculated quantities from theoretical models. The electric field in DL has been reported for different potentials and concentrations, and it is in the range E_DL_ ∈ [6.36x10^6^–1.51x10^9^] V/m, and for monovalent ions is 2.3x10^7^ V/m; choosing a value in the middle of this range, E_DL_ takes the value E_DL_ = 1.0x10^8^ V/m; even though this value of the electric field can be used to determine secondary parameters such as the surface charge density σ = E/ε, the potential Ψ_o_ will be used to calculate the secondary parameters since Ψ is the quantity more experimentally measured. Based on the Debye-Huckel theory, the potential at 25°C for monovalent ions Ψ_o_ = kT/e = 25.7 mV has been reported, and the potential drop occurs at 4.4 nm. In many situations, the potential Ψ_o_ varies in the range Ψ_o_ ∈ [10, 200] mV [14]; then, the value in the middle of the range is: Ψ_oDL_ = 100 mV = 0.1 V for DL and the potential Ψ_elec_ can be obtained by evaluating in x = λ: Ψ_oelec_ = Ψ_o_(x = λ) = Ψ_oDL_/e = 0.04 V.

The first secondary quantity that will be estimated is the force between the two charged surfaces, i.e., the interfacial force F_I_; the charges on these surfaces are on both sides of the electrode/electrolyte interface separated by a small distance of the order of λ_SL_; due to the small distance between charges of opposite signs, the attractive interfacial force F_I_ is very strong, stabilizing the entire DLC structure and producing, as mentioned, tight and orderly packing of charges near the saturation conditions; the intense force F_I_ allows to increase the charge accumulation in DL without losing stability, with a concomitant increment in the stored energy. There are several different ways to determine the interfacial force F_I_. The force between two charged surfaces depends on the electric field between the layers $F_{I}=\frac{\varepsilon }{2}AE^{2}$. Several aspects must be considered about the validity of this expression for the double-layer structure under consideration; in a cell, there are in total four charged surfaces: two couples (charged electrode)-(diffuse layer) which are in contact through the interface, i.e., these charged surfaces are in two different phases, and the electric fields in each of these phases are different; then, it is not clear the validity of this expression for calculating F_I_. Due to this, F_I_ will be calculated using the Coulomb law for the electrostatic attraction force between the electrons layer in the electrode and the ions layer in the EDL. The distance between the charged surfaces is δ = λ_SL_ + λ_e-e_ ≈ λ_SL_ = 0.2 nm; this distance is significantly smaller with respect to the distance d_min_ = √(10) = 3.16 nm between ions in the Stern layer, i.e., d_min_ it is six times larger with respect to λ_SL_.

At such small distances, the charged surfaces cannot be considered as continuous charge distributions; then, to calculate the force F_I_ between charged surfaces, the Coulomb Law was applied to each electron-ion pair multiplied by the number of pairs to get the total force F_I_; because d_min_ >> λ_SL_, only the electron-ion pairs separated by a distance λ_SL_ (first neighbors) will be considered; the reason for this is because the charge in the Stern layer produces strong shielding effect on the electric field inside the double layer reducing the contribution of layers far away from the interface; due this, the pairs separated by a distance √(λ_SL_^2^ + d_min_^2^) (second neighbors) or greater contribute with 2–3%. F_I_ can be approximated as $F_{I}=‐n(\frac{1}{4\pi \varepsilon })\frac{e^{2}}{\lambda_{\mathrm{SL}}^{2}}$ where there are $n=\frac{A}{a}=\frac{4x10^{4}\;m^{2}}{10\;{\mathrm{nm}}^{2}}=4x10^{13}$ ions in area A; the minus sign is because the force is attractive; this expression provides the minimum value for F_I_ because it only takes into account the interaction between close pairs, i.e., first neighbors; using this equation it is obtained: F_I_ = 909 N; however, taking into account the cross terms F_I_ = 1,020 N. This strong, attractive force stabilizes both charged surfaces, the whole DLC structure, and it is responsible for the very tight and orderly packing of ions in DL. This intense attraction force allows to infer the possibility of increasing the charge stored in DL without sacrificing its stability.

Even though the electric field has been experimentally measured and reported for different systems, it is possible to determine it based on the potential Ψ; this has the advantage that Ψ has been directly measured experimentally and widely reported. The electric fields E_DL_ and E_elec_ can be obtained based on the potential Ψ. Because the electric field depends on the position, their average values will be determined in DL and electrostatic regions: [0, λ] for E_DL_ and [λ, d-λ] for E_elec_; because there are two interfaces, and there is a double contribution in the middle region, the electric fields calculated from $E=‐\frac{\mathrm{d\Psi}}{\mathrm{dx}}$ has to be duplicated: $E_{DL}=2\frac{1}{2}[E_{x=0}+E_{x=\lambda }]=[\kappa \Psi_{o}+\frac{\kappa \Psi_{o}}{e}]$ = 1.368x10^8^ V/m; similarly, E_elec_ can be calculated: $E_{elec}=2\frac{1}{2}[E_{x=\lambda }+E_{x=d‐\lambda }]=[\frac{\kappa \Psi_{o}}{e}]$ = 0.368x10^8^ V/m; these values are in the range experimentally reported for the electric field; as can be observed, 80% of the electric field is in DL (in a volume of 4x10^-13^ m^3^) and 20% in elec (in a volume of 4x10^-7^ m^3^). Based on these results, it is possible to obtain the charge and charge density in DL and elec. Near a charged surface, the electric field E and surface charge density σ are related by E = σ/ε; this is a general expression for values of E close to the charged surface; then, σ_DL_ = εE_DL_; and ρ_oDL_ = σ_DL_/λ = εκE_DL_ = 4.54x10^7^ C/m^3^; for elec σ_elec_ = εE_elec_ and ρ_oelec_ = σ_elec_/d = εE_elec_/10^−3^ = 12.2 C/m^3^. From these values, it is possible to obtain the electric charge: Q_DL_ = ρ_o_Aλ = 1.82x10^-5^ C and Q_elec_ = ρ_o_Ad = 4.88x10^-6^ C; as in the electric field case, 93% of the charge is in DL and 7% in elec.

The energy stored in the double-layers was determined using $U_{\mathrm{DL}}=\frac{\varepsilon }{2}\int E^{2}\mathrm{Adx}$, where the integration limits were taken in the corresponding regions for E_DL_ and E_elec_; because E and Ψ decay rapidly with the distance, to evaluate the integral, the integrand (Ψ/x)^2^ was approximated by (Ψ/λ)^2^; the energy stored in two double-layer capacitors is: $U_{\mathrm{DL}}=2\frac{\varepsilon }{2}\int_{0}^{\lambda }\frac{\Psi^{2}}{\lambda^{2}}dV_{\mathrm{ol}}=0.432\varepsilon A\Psi_{o}^{2}\kappa$ = 3.89x10^-7^ J, and the corresponding energy density is u_DL_ = U_DL_/Aλ = 0.97 x 10^6^ J/m^3^. For $U_{\mathrm{elec}}=2\frac{\varepsilon }{2}\int_{\lambda }^{d‐\lambda }\frac{\Psi^{2}}{\lambda^{2}}dV_{\mathrm{ol}}=0.136\varepsilon A\Psi_{o}^{2}\kappa$ = 7.67x10^-9^ J, and energy density u_elec_ = U_elec_/Ad = 1.92x10^-2^ J/m^3^; the energy density in DL is seven orders of magnitude bigger with respect to elec. In a cell, three capacitors are formed: two C_DL_ at the interfaces and an electrostatic capacitor in the middle region; these capacitances are: C_DL_ = εA/2λ, and C_elec_ = εA/a = εA/(d– 2λ) ≈ εA/d. The total capacitance C_T_ is:

$$\frac{1}{C_{T}}=\frac{d}{\varepsilon A}=\frac{a}{\varepsilon A}+\frac{\lambda }{\varepsilon A}+\frac{\lambda }{\varepsilon A}$$ (6)

**Fig 3:**
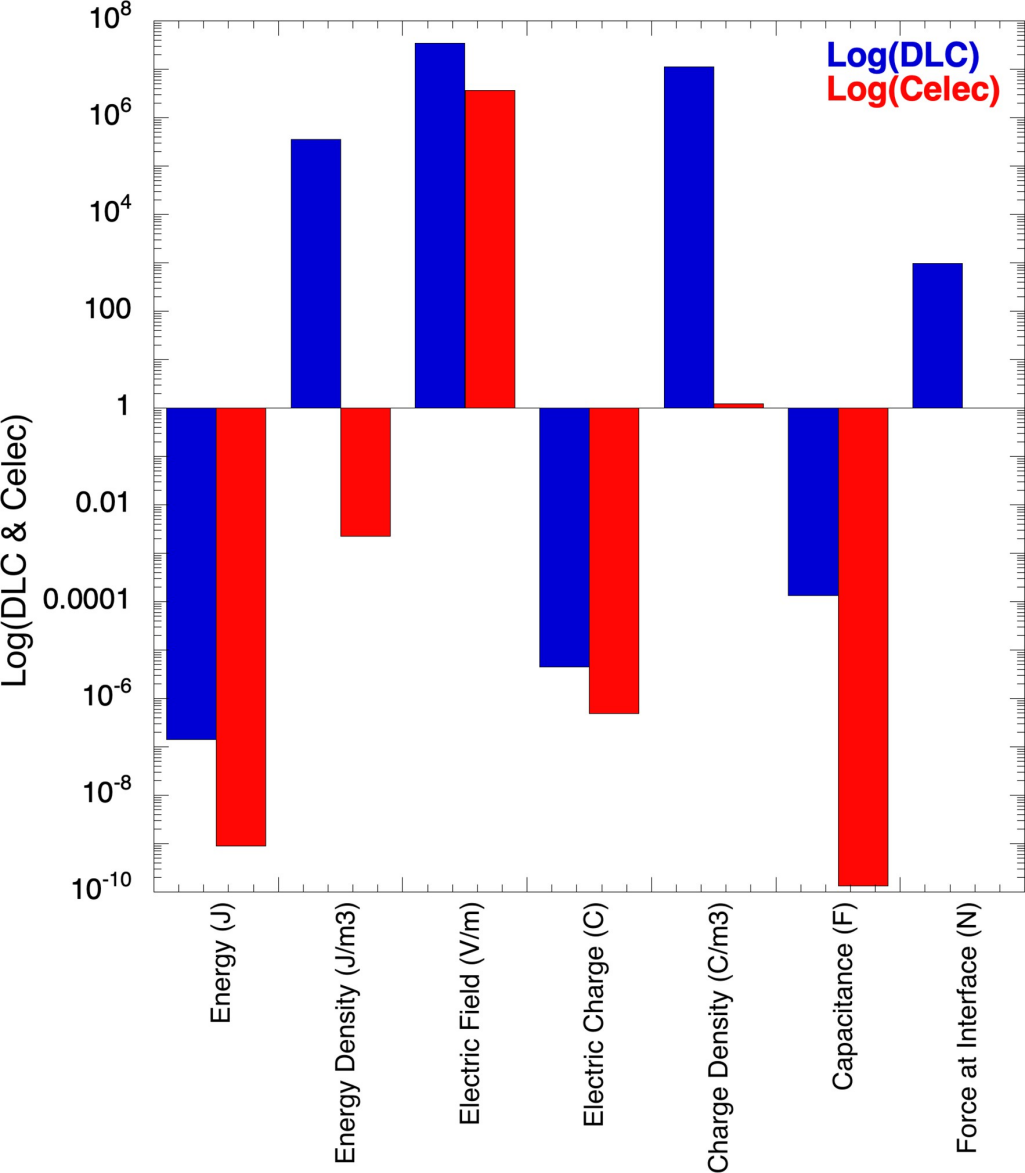
Comparative histogram of numerical values of parameters in DL and *elec* regions.

The capacitances in DL and *elec* have the values: $C_{DL}=\frac{\varepsilon A}{\lambda }$ = εAκ = 1.33x10^-4^ F and $C_{\mathrm{elec}}=\frac{\mathrm{\varepsilon A}}{\left(d‐2\lambda \right)}\approx \frac{\mathrm{\varepsilon A}}{d}$ = 1.33x10^-10^ F; these capacitances differ by six orders of magnitude. This big difference is due to the low values of the Debye length λ: all these parameters depend linearly with κ = λ^-1^ a factor in the range [10^8^, 4x10^9^]. Because Q = CV, when C increases, Q increases, that is, the amount of charge that can be stored in DL is significantly greater than in a standard capacitor: the small distance between charged surfaces, i.e., the intense attraction force F_I_ allows to store a lot of charge on DL without losing stability. These results can be compared with a standard capacitor with the same area and distance used in the previous calculations: A = 4x10^-4^ m^2^ and a separation distance of 10^−3^ m; for this standard capacitor: U = 1.66x10^-13^ J, u = 4.15x10^-7^ J/m^3^, E = 50 V/m, Q = 1.0x10^-6^ C, ρ_o_ = 16.6 C/m^3^, C = 1.33x10^-10^ F; in all cases, the parameters in the double-layer were improved by a factor of 10^6^ or higher with respect to standard capacitor. Fig 3 shows a histogram plot in the log scale of the parameter for DL and elec.

These numerical values support the mentioned that a large amount of charge can be stored in double-layer capacitors. The advantage of these estimations is to visualize potential important applications of this type of structure: DLC structures with thickness in the range of nanometers are the basic units through which it is possible to build systems to store large amounts of energy and charge; this can be achieved by connecting in series a large number of these basic capacitors. The obtained values of the parameters in DL will be discussed in relation to those obtained for the *elec* capacitor:

- **Energy:** the energy stored in a volume Vol_DL_ = Aλ = 4x10^-13^ m^3^ is U_DL_ = 3.89ex10^-7^ J; this is two orders of magnitude greater than the energy stored in volume Vol_elec_ = Ad = 4x10^-7^ m^3^: U_elec_ = 7.67x10^-9^ J; Vol_DL_ is 10^6^ times smaller than Vol_elec_; then, the energy densities are: u_DL_ = 0.97x10^6^ J/m^3^ and u_elec_ = 1.92x10^-2^ J/m^3^; the difference is a factor of 10^8^. According to these values, the DLC can store large amounts of energy.
- **Electric Charge:** The electrical charges are similar in both regions; Q_DL_ = 1.82x10^-5^ C and Q_elec_ = 4.88x10^-6^ C; however, the charge densities are significantly higher in DL with respect to elec: ρ_oDL_ = 4.54x10^7^ C/m^3^ and ρ_oelec_ = 12.2 C/m^3^, i.e., the difference is a factor of 10^6^. The DL structure is ideal to be used in the manufacture of high charge storage batteries.
- **Electric Field:** the electric field in DL is E_DL_ = 1.37x10^8^ V/m, while in elec is E_elec_ = 0.37x10^8^ V/m; this means that 80% of the electric field is in DL region: the DLC is where the electric field is stored.
- **Interfacial Force:** this is an important quantity because this attractive force holds together the two charged surfaces, providing stability to the DL structure; this interfacial force is F_I_ = 1,020 N, which is an intense force with respect to the small size of the system 4x10^-13^ m^3^. this value is considerable larger than that previously obtained, 112.5 N; the large difference is due to the small thickness of the Stern layer.
- **Capacitance:** because the thickness in the DL region is of the order of 10^−9^ m, the capacitance C_DL_ is significantly larger with respect to C_elec_: C_DL_ = 1.33x10^-4^ F and C_elec_ = 1.33x10^-10^ F. For a capacitor Q = CV, a large capacitance allows storing high amounts of charge.
- **Volume:** the DL volume is Vol_DL_ = 4x10^-13^ m^3^, while the volume for the middle region is Vol_elec_ = 4x10^-7^ m^3^.

In all determinations of double-layer parameters appear, in a natural way, the main characteristic of the DL, its thickness λ, or equivalently κ = λ^-1^; κ depends linearly on the valence and concentration Eq (5): if the valence z increases, κ increases; then, the initial assignment to basic quantities must take into account this dependence: for monovalent ions (Na^+^, K^+^) κ = κ_i_, for divalent (Sn^2+^) κ = √2κ_i_, for trivalent (Al^3+^) κ = √3κ_i_; these corrections have to be made depending on the ions present in the system.

Based on the results obtained for energy, charge, electric field, and capacitance, it is clear that DLC has important technological applications; it is possible to design devices with high output voltages and with large amounts of stored energy and charge all in a reduced space, by staking in series a considerable number of DLCs. However, this configuration brings several technological problems; even when DLC can store, by itself, large amounts of energy and charge and maintain intense electric fields and voltages, the energy must be recoverable within a specific charge-discharge voltage window; this voltage window depends on the decomposition voltage of the electrolyte produced by the electrolysis; the electrolysis voltage depends on the medium in which the ions are found in the electrolyte: for organic solvents is around 3 V.

To have a supercapacitor with high operating voltages and/or large amounts of stored energy, as required at a commercial or industrial level, a bank of many small and identical DLCs stacked in series is required. For n identical DLC capacitors C_i_ connected in series, the total voltage is V_T_ = nV_i_, and the total capacitance is $C=\frac{C_{i}}{n}$; from these expressions, it is possible to obtain a total stored energy: U_T_ = nU_i_. Both quantities, energy and voltage, increase linearly with the number of capacitors in the stack; then, with n large enough, it is possible to design devices that satisfy the voltage and/or energy requirements. However, even when this seems feasible, several technological problems must be solved. The most difficult of these is related to making and stacking n small identical DLCs: these have to be identical, with the same capacitance and internal resistance. When a stack of DLCs is overloaded, capacitors with high resistance and low capacitance are at risk of decomposition and gas production, seriously affecting the performance of the entire stack. Regardless, this is just a reproducibility issue that the industry always faces. The battery industry faces similar problems because these are made by stacking voltage cells; then, in the near future, these types of DLC devices will be quotidian.

### Chemical potential μ and chemical capacitance C_μ_

A system formed by a charged surface in contact with an electrolyte gives rise to DLC; the first theoretical model to address this problem was the Debye-Huckel, providing an analytical expression for the electric potential and charge density $\Psi (x)=\Psi_{o}e^{-\kappa x}$ and $\rho (x)=\rho_{o}e^{-\kappa x}$ where, from the Poisson equation, ρ_o_ = εκ^2^Ψ_o_. The DLC provides the main contribution to C_μ_ and, since this capacitance depends on the voltage V, this allows obtaining the relationship between C_μ_ and V. To achieve this goal, it is necessary to determine the chemical potential μ of an ions’ system in the double-layer; these ions follow the Boltzmann Distribution Law: $n=n_{o}e^{‐\mathrm{ze\Psi}/\mathrm{kT}}$. Due to long-range interaction, this system cannot be considered ideal; because the potential and charge density decay rapidly with the distance x, this is measured from the interface. The change in chemical potential Δμ_j_ of an ion j surrounded by n_i_ ions is given by $\Delta \mu_{j}=‐\frac{z_{j}^{2}q_{j}^{2}}{4\pi \varepsilon }\frac{\kappa }{1+\mathrm{\kappa a}}$ [10]; solving for κ:

$$\Delta \mu_{j}=\mu_{j}‐\mu_{o}=‐\frac{z_{j}^{2}q_{j}^{2}}{4\pi \varepsilon }\frac{\kappa }{1+\mathrm{\kappa a}}\;\mathrm{or}\;\kappa =\frac{\left(\mu_{o}‐\mu_{j}\right)}{\left[\beta ‐a(\mu_{o}‐\mu_{j})\right]}$$ (7A, 7B)

where a = d_min_, z_j_, the valence, and q_j_ the charge of the ions, and β = z_j_q_j_^2^/4πε.

At the end of the Chemical Potential μ and Chemical Capacitance C_μ_ section, an analytical expression will be obtained between the chemical capacitance C_μ_ and the output voltage and the mordant concentration [%Al^3+^]; cochineal and brazilwood were used as dyes and alum as mordant. The chemical capacitance C_μ_ is defined as $C_{\mu }=e^{2}\frac{\partial n_{i}}{\partial \mu_{i}}$ where μ_i_ is the internal chemical potential. Using the Boltzmann Distribution Law $n_{i}(\mu )=n_{\mathrm{oi}}e^{\mu /\mathrm{kT}}$ and the definition of C_μ_, it is easy to obtain: $C_{\mu }=\varepsilon \kappa^{2}$; C_μ_ depends quadratically on the more important parameter of the double layer: κ. Using Eqs (7A and 7B), defining the output voltage V = Ψ_o_−Ψ measured from the interface and assuming, as in the Debye-Huckel approximation, that the potential Ψ is small, zeΨ << kT, i.e., Ψ = Ψ_o_[1 - κx + …], the Boltzmann Distribution Law can be written as:

$$n_{i}=n_{\mathrm{oi}}e^{+(\frac{q_{i}}{\mathrm{kT}})V}$$ (8)

which is more appropriate for this calculation. From the definition of C_μ_, it is possible to write $C_{\mu }=e^{2}(\frac{\partial n_{i}}{\partial \mu_{i}})=e^{2}(\frac{\partial n_{i}}{\partial \kappa })(\frac{\partial \kappa }{\partial \mu })$; the quantity $\left(\frac{\partial \kappa }{\partial \mu }\right)$ can be obtained using Eq (7B): $(\frac{\partial \kappa }{\partial \mu })=\frac{\beta }{{\left[\beta ‐a(\mu_{o}‐\mu )\right]}^{2}}$, while the factor $\left(\frac{\partial n_{i}}{\partial \kappa }\right)$ can be obtained using Eq (8) $(\frac{\partial n_{i}}{\partial \kappa })=n_{o}\gamma \Psi_{o}xe^{‐\gamma \Psi_{o}}e^{+(\frac{q_{e}}{\mathrm{kT}})V}$. Using these expressions, the final form for C_μ_ is:

$$C_{\mu }=C_{o\mu }e^{+(q_{e}/\mathrm{kT})V}\;\mathrm{or}\;{\mathrm{Ln}\;C}_{\mu }=b+\mathrm{mV}$$ (9A, 9B)

where $\beta =\frac{q_{e}^{2}}{4\pi \varepsilon }$, a = d_min_, $C_{o\mu }=\frac{(\frac{q_{e}^{2}}{4\pi \varepsilon })n_{o}(\frac{q_{e}\Psi_{o}}{kT})e^{‐2q_{e}\Psi_{o}/kT}}{{\left[\beta ‐a(\mu_{o}‐\mu )\right]}^{2}},\;b=\mathrm{Ln}(C_{o\mu })$, m = q_e_/kT. Eq (9A) predicts that the chemical capacitance depends exponentially on the voltage; this equation is the same as that reported by Bisquert & Fabregat, [15]. The experimental results report that the logarithm of C_μ_ vs V is a sigmoid function where the middle section, of such sigmoid function, is a straight line identical to Eq (9B) with a positive slope: in this region C_μ_ dominates the capacitance. The dependence of C_μ_ on temperature has been experimentally measured in the range 0 to 60°C and reported by Bisquert & Fabregat, [16]; it coincides with that obtained here: m = q/kT.

**Fig 4:**
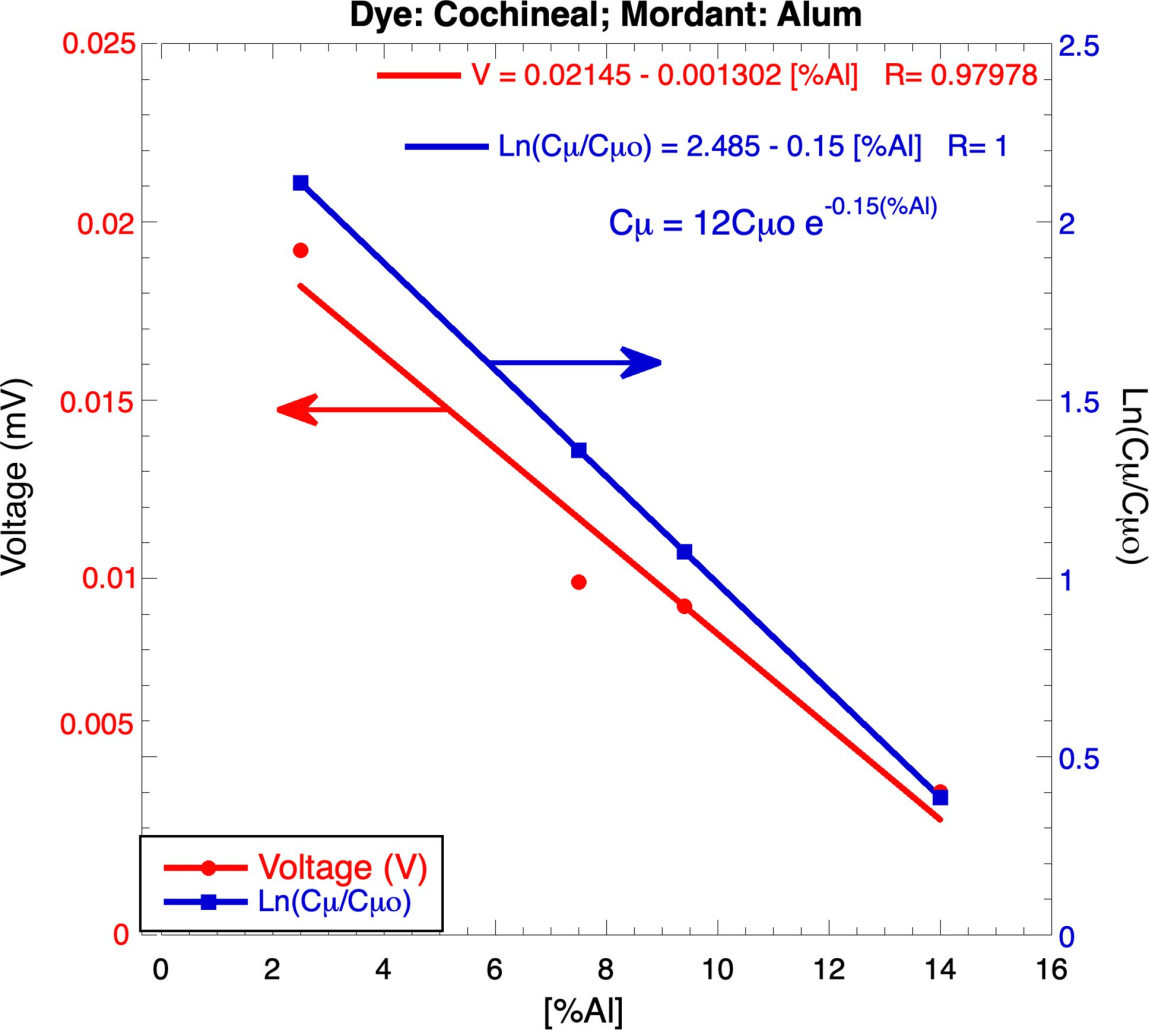
Dependence of: In red) the output voltage V, and in blue) Ln(C_μ_/C_μo_), as a function of [%Al], obtained from Eq (10) for cochineal-alum samples.

Using Fig 1B, it was possible to obtain the dependence of the output voltage as a function of the [%Al^3+^]; Fig 4 shows, in red a linear dependence of voltage with [%Al]: V = 0.021–0.0013[%Al^3+^] with R = 0.97978; based on this equation, it was possible to obtain the dependence of Ln (C_μ_) as a function of [%Al^3+^] for cochineal-alum samples as shown in blue; using this equation with Eq (9A) is obtained

$$\frac{C_{\mu }}{C_{\mu 0}}={12e}^{‐0.15[\%\mathrm{Al}]}$$ (10)

In a semi-log space this equation is a straight line shown in blue (Fig 4). Unlike the graph reported by Bisquert et al. [16], where the relationship Ln(C_μ_) vs V has a positive slope, in this case, the slope is negative. As mentioned, the Al^3+^ ions are very small with high charge and move rapidly, producing decaying profiles in all cases. These profiles reach, in short times, their final voltage values; then, if the concentration of Al^3+^ ions increases, V_i_ increases, and V decreases, i.e., they have an inverse relationship: the slope is negative for the line shown in red in Fig 4.

### Mathematical model of unstable oscillations

Because the cell was suddenly illuminated, the output voltages show unstable oscillations on top of the signals; due to this, a theoretical model based on thermodynamics was developed to fit these oscillations; in addition to providing fit to the experimental data, this model provides information regarding the mechanisms involved in these unstable oscillations. It has been reported a mathematical model to describe the unstable oscillations found on top of the output voltage profile when the photocell is exposed to an abrupt dark-to-light transition [17]. This model is based on thermodynamics where there are two forces working in opposition. During the dye-photon interaction, electrons are released from the dye, leaving it with a net positive charge; these positive charges produce stretching of the dye molecules, which has an elastic recovery force f_elas_. The stretched molecules can overlap each other, and this overlapping results in a local increment in the segment concentration given place to an osmotic pressure f_osmotic_; this force tries to separate them to reduce the concentration gradient; the forces f_elas_ and f_osmotic_ are responsible for the oscillations in the output voltage; finally, the released electrons are recovered when the circuit is completed and the cell backs to its initial stage. The model presented here is based on the Flory-Krigbaum theory [18], and it was developed to study the stability of colloids. This model allows obtaining an appropriate expression to fit the oscillations present on the profiles: x(t) = x_o_/[1+a_x_sin^2^(ω_x_t+δ_x_)]; this term has to be included in Eqs (4A and 4B):

$$V(t)=V_{o1}e^{-t/\tau_{1}}{+V}_{o2}e^{-t/\tau_{2}}+\frac{x_{o}}{\left[1+a{sin}^{2}(\omega t+\delta )\right]}+\mathrm{BL}$$ (11A)

$$V(t)={(1‐V}_{o1}e^{‐t/\tau_{1}})+{(1‐V}_{o2}e^{‐t/\tau_{2}})+\frac{x_{o}}{\left[1+a{sin}^{2}(\omega t+\delta )\right]}+\mathrm{BL}$$ (11B)

where Eq (11A) corresponds to a decaying profile and Eq (11B) to a rising one.

The shape of the output signal od DSSC is important when these cells are connected to non-linear electrical charges, for example to inverter electronic circuits DC-AC [19]. When this DSSC voltage has harmonic distortion or its shape is unpredictable, it could have high content of harmonics of different orders that could affect the operation and performance of DSSCs and the connected non-linear charges. It is also possible that due to variations in the operating temperature of the DSSC, its performance is affected, since due to the type of ions present in the electrolyte, the equivalent resistive and capacitive circuits inside the cells they would be changing their conductance or capacitance and would modify the output impedance or resistance of the DSSC [20], as well as the charge constant (τ) of the double layer capacitors and charge transfer resistances, that form within this type of cells and could influence to transfer the maximum generated power by the DSSC itself to an electrical charge connected to its output. In this work the tests on the DSSC were carried out at a temperature of 25°C.

## Experimental

### Materials, fabrication and characterization

Solar cells were manufactured as in Ref [21] using cochineal and brazilwood as natural dyes; additionally, four additives were included: two mordant, alum and Sn, and two brighteners, sodium metasilicate and cremor tartar. Five different concentrations of these four additives were prepared: C1 = 2.5, C2 = 7.5, C3 = 9.4, C4 = 14.0, and C5 = 19.0, all in mg/mL. The fabrication was done according to Ref [21], adjusting the thickness of the mesoporous to 34.7 microns. The electric characterization was done using a multimeter connected to the computer.

## Results and discussion

Fig 1A–1D show four sets of voltage profiles using two dyes, cochineal and brazilwood, with several additives, metasilicate, alum, and cremor tartar, at different concentrations (C1-C5); Sn-based samples were prepared and analyzed, the results are reported but profiles are not. In these sets of profiles, some of them are rising while others are decaying; this can be explained by analyzing the times τ_i_. Eq (1) provides an expression for the arrival time τ_i_ of the ions to EDL; in this expression, the dependence on q_i_ and R_i_ is due to the electric and viscous forces, while the dependence on n_i_ is due to the blocking effect. The added ions are Al^3+^ (alum), Sn^2+^, Na^+^ (metasilicate), and K^+^ (cremor tartar).

In a time-concentration space, Eq (1) τ_i_ ~ C_i_R_i_/q_i_ corresponds to straight lines passing through the origin Fig 2; there are four lines, one for each ion. This figure shows a correlation between the shape of the output voltage profiles, arrival times τ_i_, and ions concentration C_i_; the vertical axis was divided in rising profiles (τ_i_ > 15) and decaying profiles (τ_i_ < 14), meaning that, for cell prepared as reported, the threshold was between 14 and 15; for Al^3+^ and Sn^2+^ these straight lines do not cross the threshold, while for Na^+^ and K^+^, part of these two lines are in the rising zone and the other part in the decaying zone; as can be seen, in these cases, it is the concentration that decides whether the profile is rising or decaying (Fig 2): the shape of the profiles strongly depends on the type and concentration of ions; there is a small dependence on the type of dye since it is this that emits the conduction electrons; then, the discussion will be based on the ions’ characteristics. The ions concentrations are: C1 = 2.5, C2 = 7.5, C3 = 9.4, C4 = 14.0, C5 = 19.0, all in mg/mL. In Fig 2, the rising profiles will be indicated in **blue**, the decaying ones in **red**, and the flat ones in **black**. Each ion will be analyzed independently.

- Na^+^: this is an ion of intermediate size (R_Na_ = 0.99 Å) and small charge; the arrival times τ_i_ are **2.48, 4.95, 9.90, 14.85, 19.80**; at low concentrations, C1-C3, they move fast forming EDL in short times, increasing E_i_, reducing E_T_ and V and producing a decaying profile; at C4 the shape is flat (no rising and no decaying); at C5, the blocking effect reduces the ions mobility, delaying their arrival to EDL and allowing electrons to reach the electrode to increase the output voltage, producing a rising profile. The transition decaying-rising occurs for values between 14 and 15.
- K^+^: this is an ion of intermediate size (R_Na_ = 1.38 Å) and small charge; the times τ_i_ are: **3.45, 6.90, 13.80, 20.70, 27.60**; at low concentrations, C1-C3, they move fast forming EDL in short times and producing a decaying profile; at C4-C5, the blocking effect reduces the ions mobility, delaying their arrival to EDL and producing a rising profile. As in the former case, the transition decaying-rising occurs for values between 14 and 15.
- Al^3+^: this is a small ion (0.54 Å) with a high charge; the times τ_i_ are **0.45, 0.90, 1.80, 2.70, 3.60**; these ions move quickly and also quickly reach the EDL, reducing the output voltage and producing, in all cases, decaying profiles; as can be seen, these values are lower than 14.
- Sn^2+^: this is a small ion (0.71 Å) with a high charge; the times τ_i_ are **0.89, 1.78, 3.55, 5.33, 7.10**; as in the case of Al^3+^, these ions move fast, also reaching rapid the EDL reducing the output voltage and producing decaying profiles; these values are lower than 14. When the values of τ_i_ are lower than 14, the ions involved give rise to decaying profiles, while when the values of τ_i_ are higher than 15, the ions involved give rise to rising profiles.

**Fig 5:**
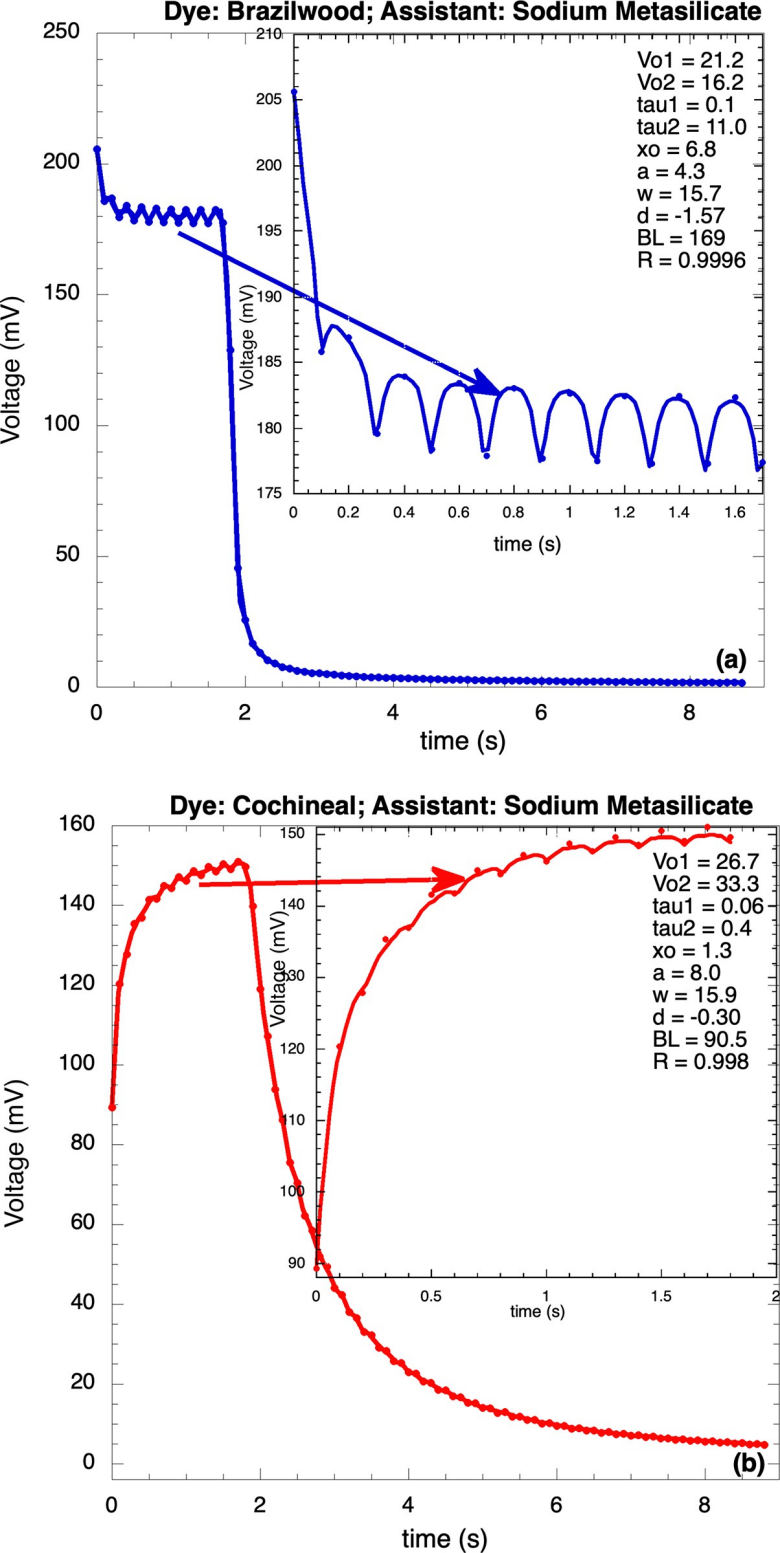
Voltage profiles of a) brazilwood and b) cochineal using metasilicate as brightener; the oscillations were fitted using Eqs (11A and 11B).

In Fig 5A and 5B are reported oscillations on decaying (Fig 5A) and rising (Fig 5B) profiles; the continuous curve the fitting using Eqs 11A and 11B; the correlation coefficients show that the fits were good (R = 0.99962 and 0.99929), supporting the validity of the model. The results of the fitting parameters of these profiles are, for Fig 5A: V_o1_ = 21.25 mV; V_o2_ = 16.18 mV; τ_1_ = 0.096 s; τ_2_ = 11.02 s; x_o_ = 6.78 mV; a = 4.33; ω = 15.82 s^-1^; δ = -1.57; and BL = 169.4 mV. For Fig 5B: V_o1_ = 27.0 mV; V_o2_ = 34.0 mV; τ_1_ = 0.05 s; τ_2_ = 0.4 s; x_o_ = 1.5 mV; a = 2.0; ω = 15.7 s^-1^; δ = 0.02; and BL = 90.0 mV. The amplitudes of the unstable oscillations depend on the dye and additives: the cells made with cochineal-metasilicate and brazilwood-alum showed oscillations with low amplitude; this is important because the oscillations can affect, negatively, the equipment connected to the cells when they are subject to abrupt changes in lighting. The non-linear model sketched here allows an understanding of the factors involved in forming the unstable oscillations. The oscillation frequency is practically constant for all samples ω = (15.72 ± 0.2) s^-1^ (f = 2.51 Hz).

As mentioned, the numerical values assigned to basic quantities were according to the values reported in the literature; some were measured experimentally, and others were obtained using a theoretical model such as Debye-Huckel. Using these values, it was possible to estimate, numerically, various important parameters of DLC: charge, energy, capacitance, interfacial force, and electric field. Before illumination, C_elec_ = εA/d, U = 0, E = 0, and ρ_o_ = 0 because there are no charges, stored energy, DL, and electric field. However, when the cell is illuminated, the produced electrons give rise to electric fields, energy storage, and DL formation. These estimations provide interesting results, such as the possibility of having devices that can deliver high voltages and considerable amounts of energy in small volumes just by stacking a significant number of DLCs in a series configuration. A simple capacitor containing an electrolyte inside has two DLCs at the interfaces, and one C_elec_ in the central zone. As reported in the Numerical Estimation of DSSC Parameters section, the stored energy in DL is U_DL_ = 3.89x10^-7^ J, and in the middle region U_elec_ = 7.67x10^-9^ J; this means that 98% of the energy is in DL and 2% in elec; DLCs have a structure particularly suitable for storing large amounts of energy and charge in small volumes for later recovery. The energy densities for DL and elec are u_DL_ = 3.07x10^6^ J/m^3^ and u_elec_ = 3.03x10^-2^ J/m^3^: there is a difference of eight orders of magnitude between these values due to the small DL volume (≈ 10^−13^ m^3^). The average electric field was obtained from $E=‐\frac{\mathrm{d\Psi}}{\mathrm{dx}}$, resulting: E_DL_ = 6.84x10^7^ V/m, and E_elec_ = 7.36x10^6^ V/m, i.e., 90% of the electric field is in DL and 10% in elec.

The number of ions in the Stern layer is strongly related to the number of electrons in the electrode; this is due to the strong attractive electrostatic force between electrons and ions and the high mobility of ions in the electrolyte; the distance between electrons and ions is of the order of λ_SL_ = 0.5 nm which is significantly less than the ion-ion distance d_min_ = 3.16 nm; then, the attractive force is significantly greater than the ion-ion repulsive force (a factor of 40). Using the Coulomb Law it was possible to estimate the force between the electrons in the electrode and the ions in EDL, resulting in F_I_ = 1,020 N; this is a strong force for a small volume (Aλ_SL_). With respect to the electric charge Q, something similar to the electric field happens: 79% of the electric charge is in DL and 21% in the middle region. To determine the electric charge and charge density, it was used the relationship E = σ/ε valid near the charged surface; then, σ_DL_ = εE_DL_ and ρ_oDL_ = εκE_DL_ = 4.54x10^7^ C/m^3^ and σ_elec_ = εE_elec_, then ρ_oelec_ = εE_DL_/10^3^ = 12.2 C/m^3^; the charge density in DL is six orders of magnitude greater than in elec, i.e., practically all charge is in DL. From these values for the charge densities, it is possible to determine the charges: Q_DL_ = 1.82x10^-5^ C and Q_elec_ = 4.88x10^-6^ C.

From $C_{\mu }=e^{2}(\frac{\partial n_{i}}{\partial \kappa })(\frac{\partial \kappa }{\partial \mu })$ and using the expressions: $(\frac{\partial \kappa }{\partial \mu })=\frac{\beta }{{\left[\beta ‐a(\mu_{o}‐\mu )\right]}^{2}}$, and $(\frac{\partial n_{i}}{\partial \kappa })=n_{o}\gamma \Psi_{o}xe^{‐\gamma \Psi_{o}}e^{+(\frac{q_{e}}{\mathrm{kT}})V}$, it was obtained $C_{\mu }=C_{o\mu }e^{+(q_{e}/\mathrm{kT})V}$ or, in a log space, ${\mathrm{Ln}\;C}_{\mu }=b+(q_{e}/\mathrm{kT})V$ (Eq 9A and 9B) i.e., C_μ_ depends exponentially on V. This expression, together with the temperature dependence (q/kT), resulting equal to those obtained by Bisquert et al. [16]. Using the information reported in Fig 1B, it was also possible to obtain a relationship between C_μ_ and [%Al^3+^]: $\frac{C_{\mu }}{C_{\mu 0}}={12e}^{‐0.15[\%\mathrm{Al}]}$; this relation is reported in Eq (10). Respect to unstable oscillation, the mathematical model presented here fits well the voltage profiles containing oscillations.

The numerical estimation of characteristic parameters of DLC opens many potential applications for this type of capacitor; the amount of energy and charge that can be stored in DLC is overwhelmingly greater respect to electrostatic capacitors; the structure of these capacitors (charged surface-electrolyte) allows a very appropriate synergy that produces, on the one hand, a very stable structure and, on the other, a great capacity to store, in very small regions, large amounts of energy and charge. The first idea that comes to mind is the manufacture of super-batteries with high capacity and stability; the great sensitivity of this type of capacitors also has potential applications in medicine.

## Conclusions

The shape of the output voltage profiles depends on comparing the arrival times of electrons τ_e_ and ions τ_i_ to their respective destinations; the times τ_i_ depend on the size, charge, and concentration of the added ions introduced by mordant and brighteners. Large ions of small charge and high concentration move slowly, delaying their arrival at EDL; this allows more electrons to reach the electrodes, increasing the output voltage and leading to a rising profile. On the other hand, small ions with high charge and low concentration move quickly, reaching the double layer, which reduces the output voltage, leading to a decaying profile. To fit the experimental data containing unstable oscillations, a model was required to provide the appropriate mathematical expression to carry out the corresponding fitting; the model reported here allows for determining the factors that control these oscillations. The estimation of important parameters that characterize DLC allows to say that this type of double-layer capacitor can store large amounts of energy and charge; these results show that, as a common factor, all these parameters depend linearly on κ (= 10^9^ m), the inverse of λ; this dependency allows to affirm that double-layer capacitors can store large amounts of energy at intense electric field; the numerical estimates were made at energy, electric field, charge, and capacitance. Additionally, it was possible to determine the dependence of C_μ_ on voltage V which is exponential $C_{\mu }=C_{\mathrm{o\mu}}e^{(\frac{q}{\mathrm{kT}})V}$; using a similar methodology, it was also possible to obtain a relationship between C_μ_ and Al^3+^ concentration which is also exponential $C_{\mu }={12C}_{\mathrm{\mu o}}e^{‐0.15(\%\mathrm{Al})}$. There are many technological applications of DLC due to its important energy storage properties in small volumes; the obvious application is in the manufacture of super-batteries.

## Supporting information

S1 Data Fig 1A data. (XLSX)

S2 Data Fig 1B data. (XLSX)

S3 Data Fig 1C data. (XLSX)

S4 Data Fig 1D data. (XLSX)

S5 Data Fig 5 data. (XLSX)
